# Acute scoliosis as an unusual presentation of pneumonia: A case report

**DOI:** 10.1097/MD.0000000000010580

**Published:** 2018-06-15

**Authors:** Rajan Atwal, Charles Stewart

**Affiliations:** Department of Paediatric Emergency, Chelsea and Westminster Hospital, Chelsea, London.

**Keywords:** paediatrics, pneumonia, scoliosis

## Abstract

We describe the unique case of a child with pneumonia presenting with acute scoliosis and abdominal pain, without any typical features of the disease.

A 10-year-old girl presented to the emergency department on 3 consecutive days with right-sided abdominal pain. There were no associated features, in particular, no fevers or respiratory symptoms. On the first 2 presentations, observation,examination, and blood test findings were unremarkable. Chest x-ray and abdominal ultrasound were also normal. On the third presentation a marked scoliosis was noted and abdominal examination revealed right-sided tenderness with rebound. The patient was admitted and a computed tomographic scan of the abdomen arranged. Unexpectedly, this revealed a right lower lobe pneumonia and associated pleural effusion. Despite treatment, the parapneumonic effusion enlarged rapidly and she developed respiratory distress, necessitating transfer to a tertiary centre.

The diagnosis of pneumonia can be challenging because of a lack of respiratory signs, the masking of systemic features by antipyretic effects of first-line analgesics, and a high rate of false-negative chest radiographs. The development of acute scoliosis should lead the clinician to strongly consider pneumonia in such circumstances.

## 1 Introduction

Pneumonia is an important public health condition and in 2015 accounted for 16% of all deaths of children <5 years’ old globally.^[1]^ It is a clinical diagnosis typically made from a combination of infective and respiratory signs and symptoms. We describe the case of a child who presented with acute scoliosis and abdominal pain without any typical features of the disease.

## 2 Case report

A 10-year-old girl presented with a 2-day history of constant right-sided flank pain with intermittent episodes of increased intensity. Movement and laying supine exacerbated the pain, whereas some relief was found with paracetamol. There was no trauma to the area, but the patient reported playing netball shortly before the pain started. There was no associated nausea, vomiting, or change in bowel or urinary habit. There was no fever and no symptoms of cough or breathing difficulties.

The patient had no significant medical history and immunisations were up to date. On examination, she was apyrexial with a heart rate of 110 beats per minute, oxygen saturations of 99% on room air and respiratory rate of 22 breaths per minute. She was warm and well perfused with a central capillary refill time of <2 seconds. She appeared distressed when laying down reporting worsening pain; however, the abdomen was soft and non-tender to palpation. Systemic examination was otherwise unremarkable.

Urine analysis showed no evidence of infection and blood tests revealed a C-reactive protein (CRP) of 23 mg/L and a normal full blood count with white cells of 13.4 × 10^9^ cells/L. Liver function tests, urea and electrolytes, and venous blood gas were also normal.

With normal observations, examination and investigation results and a good response to analgesia in the department, the patient was discharged with a planned review.

**Figure 1:**
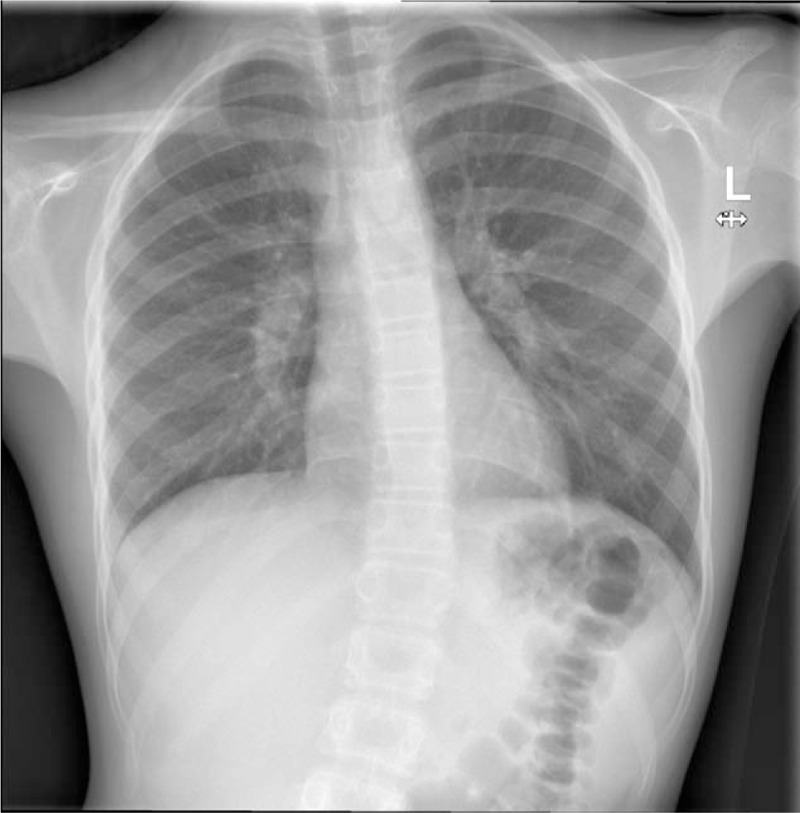
Normal chest radiograph on first presentation to hospital.

On re-assessment the following day, she continued to complain of significant pain on the right side of the abdomen with similar features on examination. Owing to the persistence of symptoms, chest radiography (Fig. 1) and abdominal ultrasound were performed. Both were reported as normal. With no surgical or medical cause of the pain identified, it was deemed musculoskeletal in origin, associated with playing netball. She was discharged with advice to take regular analgesia and to return if symptoms were not settling.

The patient represented the following day looking unwell. She appeared very pale with dry lips and walked slowly, hunched over. She was tachycardic at 130 beats per minute and tachypnoeic with a respiratory rate of 32 breaths per minute. Oxygen saturations, temperature, and blood pressure were normal at 98%, 36.8^0^C, and 119/72 mmHg respectively. She stood with her right hip and knee slightly flexed, with her trunk deviated to the left giving her a marked scoliosis. Musculoskeletal examination of the spine, hips, and knees was unremarkable, but it was noted that abdominal pain worsened on flexion of the hip against resistance. Cardiovascular and respiratory examinations were normal. She was unable to lie supine because of severe pain and was examined in the semirecumbent position. Active distension of the abdomen and coughing further exacerbated the pain and she was found to have guarding with rebound tenderness in the right flank and hypochondrium. An intravenous cannula was inserted and intravenous fluids, paracetamol, and ibuprofen were commenced while awaiting a surgical review for her acute abdomen. Concurrent orthopedic opinion was also sought because of the new-onset scoliosis.

**Figure 2:**
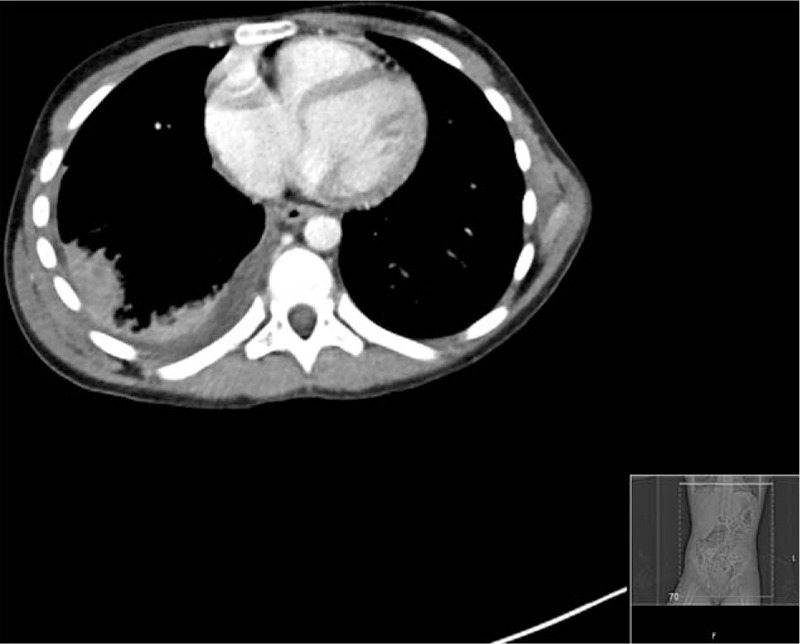
Transverse slice of computed tomography scan of abdomen and pelvis showing right lower lobe consolidation.

**Figure 3:**
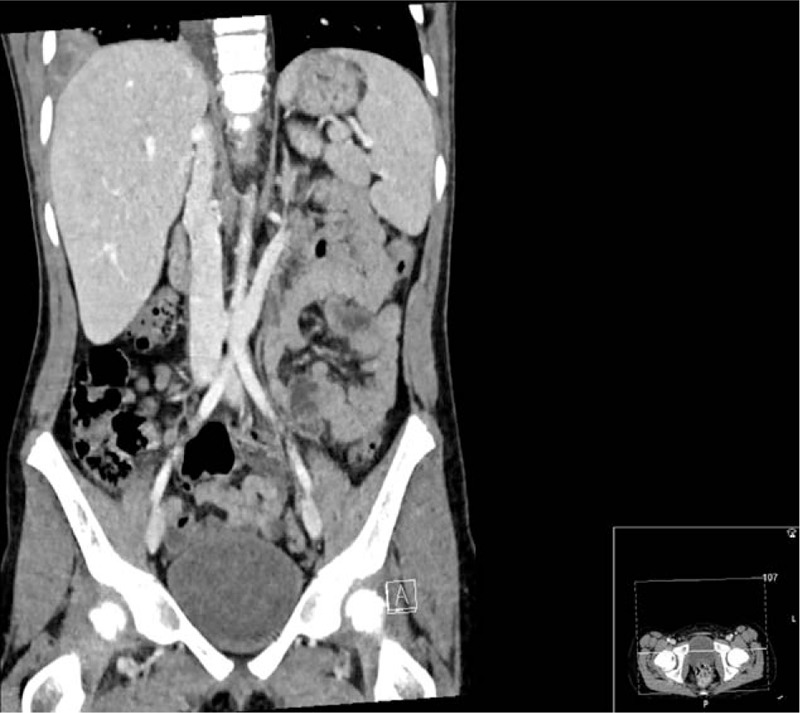
Coronal slice of computed tomography scan of abdomen and pelvis showing right lower lobe consolidation.

After treatment the patients’ pain was significantly reduced, pallor had resolved and her observations normalised. Abdominal examination at the time of surgical review was normal, but because of repeated presentations with abdominal pain and a rising CRP (93 mg/L) and white cell count (13.9 × 10^9^ cells/L) the patient was admitted and a computed tomography (CT) scan of the abdomen and pelvis was arranged. Orthopedic review did not yield any acute orthopedic concerns regarding the scoliosis and they agreed with the plan for abdominal imaging. The CT scan failed to reveal an abdominal cause for her pain, but to our surprise showed an organizing right lower lobe pneumonia with a small effusion (Figs. 2 and 3).

**Figure 4:**
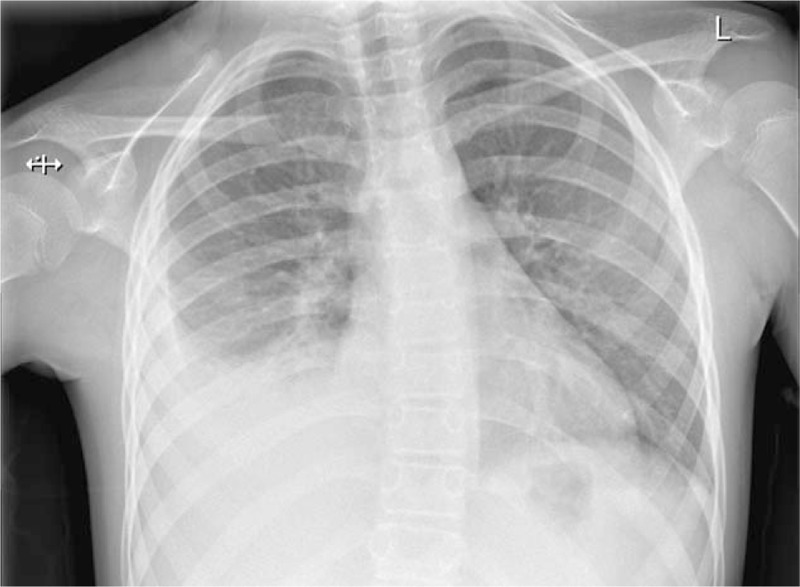
Chest radiograph on day 3 of hospital admission showing a moderate right sided pleural effusion. Note the scoliosis, with the convexity of the spine pointing away from the side of the effusion.

**Figure 5:**
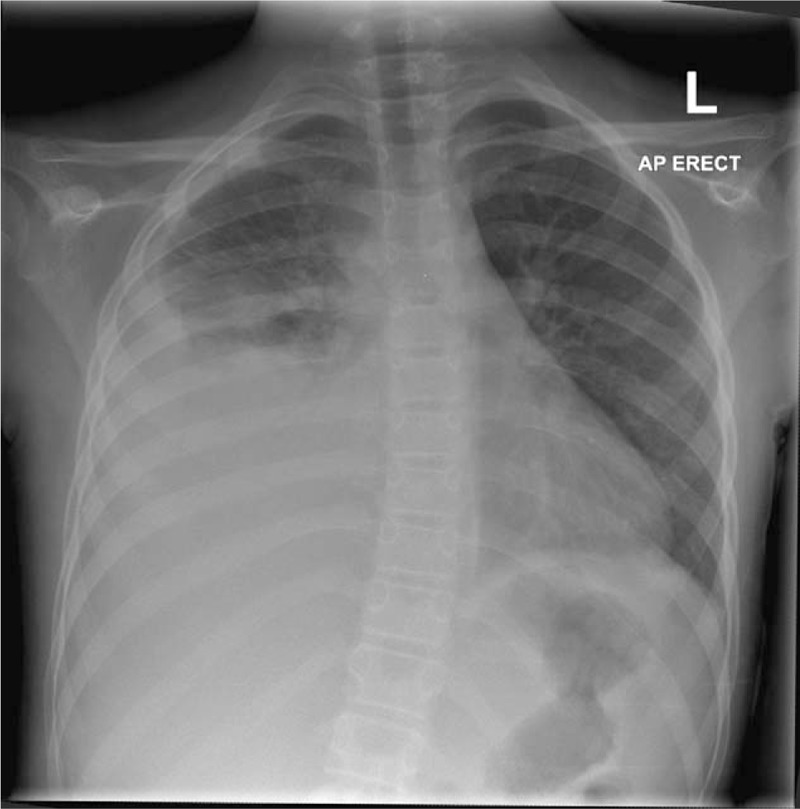
Chest radiograph on day 6 of hospital admission showing a large right-sided pleural effusion.

The patient was started on intravenous antibiotics for the thus far silent pneumonia. Despite treatment during the next few days, the pneumonia progressed and she developed a large parapneumonic effusion (Figs. 4 and 5). On the 6th day of hospital admission, she deteriorated rapidly with signs of sepsis and respiratory distress. This was accompanied by a further rise in CRP (351 mg/L) and white cell count (17.3 × 10^9^ cells/L). She was stabilized and then transferred to a tertiary hospital for further management.

### 2.1 Follow-up and outcomes

At the tertiary center, a chest drain was inserted and the patient was treated with intrapleural urokinase therapy and intravenous antibiotics. She was discharged after a 2-week stay.

With the complete resolution of the pneumonia and scoliosis, the patient made a graduated return to school and at 4 months post discharge was almost back to full-time attendance.

## 3 Discussion

Pneumonia is the single largest infectious cause of death in children worldwide and in the United Kingdom 12.2 per 10 000 children aged 0 to 16 years per annum are admitted to hospital.^[1,2]^ It is therefore a very important disease to diagnose and treat. Children typically present with ≥1 features of fever, tachypnoea, dyspnoea, respiratory distress, cough, wheeze, or chest pain.^[2]^ This is not an exhaustive list, however, and clinicians need to be open to atypical presentations.

We report the case of a 10-year-old girl with pneumonia who presented with abdominal pain which, although a well-recognized symptom in children with pneumonia, is often experienced in combination with fever and/or cough.^[3,4]^ She did not have any of the above symptoms or signs, although the antipyretic effects of first-line analgesics may have masked systemic features. Nevertheless, we did consider pneumonia as a diagnosis and requested a chest radiograph accordingly. The radiology report was negative and, as the patient remained free of features of pneumonia, other differential diagnoses were explored for this girl's symptoms.

We are heavily reliant on plain chest films in the Pediatric Emergency Department to aid in the diagnosis of pneumonia despite the test's accuracy being reported as quite variable.^[5]^ Studies demonstrate false-negative reporting rates of up to 27% in the presence of CT diagnozed pneumonias.^[6,7]^ Ultrasonography appears to be more accurate than plain films, although the criterion standard remains CT. Despite this, without a strong pretest suspicion, many clinicians would be reluctant to request this test because of the associated exposure to radiation.

The development of scoliosis eventually led to the correct diagnosis of pneumonia, albeit fortuitously, as an incidental finding on CT scanning. An acute presentation in the emergency department of scoliosis would typically prompt the clinician to look for evidence of local infection (e.g., osteomyelitis of vertebral body or discitis), trauma, or malignant infiltration.^[8–10]^ Paraspinal muscular spasm secondary to injury, appendicitis, or pyelonephritis are also recognized causes of scoliosis.^[11,12]^ What seems to be less well known about in the emergency setting is the association of scoliosis and pleural infection (parapneumonic effusion and empyema) in children. British Thoracic Society guidelines list scoliosis as one of the examination findings that suggest pleural effusion in children, but interestingly not in the adult guidelines.^[13,14]^ Although they state it is transient and no specific treatment is required, resolution must be confirmed to exclude the other causes.

A retrospective study by Mukherjee et al^[15]^ of 122 children hospitalized with pleural infections showed that thoracic scoliosis was present in 46% (n = 56) on admission and in 71% (n = 87) during some stage of the illness. The scoliosis had no association with inflammatory markers, size or type of effusion.^[15]^ All patients in the study had the convexity of the spine pointing away from the affected lung, which is consistent with our case findings (Figures 1, 4 and 5).^[15]^ This is thought to reflect splinting of the chest wall to reduce pain.^[13,16]^ A case report by Malhotra et al^[12]^ refers to a 3-year-old child with pneumonia presenting with acute scoliosis; however, on examination they were found to have reduced air entry on the left base with positive x-ray findings. What makes our case of pneumonia unique is the lack of any supporting clinical or radiological evidence of the disease.

## 4 Conclusions

The diagnosis of pneumonia can be challenging because of a lack of respiratory signs, the masking of systemic features by antipyretic effects of first-line analgesics, and a high rate of false-negative chest radiographs. The development of acute scoliosis should lead the clinician to strongly consider pneumonia in such circumstances.

## Author contributions

**Conceptualization:** Charles Stewart and Rajan Atwal.

**Project administration:** Charles Stewart.

**Supervision:** Charles Stewart.

**Writing – original draft:** Rajan Atwal.

**Writing – review & editing:** Charles Stewart and Rajan Atwal.
